# Transmission dynamics of rabies virus in Thailand: Implications for disease control

**DOI:** 10.1186/1471-2334-5-52

**Published:** 2005-06-29

**Authors:** Jessada Denduangboripant, Supaporn Wacharapluesadee, Boonlert Lumlertdacha, Nipada Ruankaew, Wirongrong Hoonsuwan, Apirom Puanghat, Thiravat Hemachudha

**Affiliations:** 1 Department of Biology, Faculty of Science, Chulalongkorn University, Bangkok, Thailand; 2 Molecular Biology Laboratory for Neurological Diseases, Chulalongkorn University Hospital, Bangkok, Thailand; 3 Queen Saovabha Memorial Institute, Thai Red Cross Society, Bangkok, Thailand; 4 Department of Biology, Faculty of Science, Chulalongkorn University, Bangkok, Thailand; 5 Department of Livestock Development, Ministry of Agriculture, Bangkok, Thailand; 6 Department of Disease Control, Ministry of Public Health, Nonthaburi, Thailand; 7 Molecular Biology Laboratory for Neurological Diseases, Chulalongkorn University Hospital, Bangkok, Thailand

## Abstract

**Background:**

In Thailand, rabies remains a neglected disease with authorities continuing to rely on human death statistics while ignoring the financial burden resulting from an enormous increase in post-exposure prophylaxis. Past attempts to conduct a mass dog vaccination and sterilization program have been limited to Bangkok city and have not been successful. We have used molecular epidemiology to define geographic localization of rabies virus phylogroups and their pattern of spread in Thailand.

**Methods:**

We analyzed 239 nucleoprotein gene sequences from animal and human brain samples collected from all over Thailand between 1998 and 2002. We then reconstructed a phylogenetic tree correlating these data with geographical information.

**Results:**

All sequences formed a monophyletic tree of 2 distinct phylogroups, TH1 and TH2. Three subgroups were identified in the TH1 subgroup and were distributed in the middle region of the country. Eight subgroups of TH2 viruses were identified widely distributed throughout the country overlapping the TH1 territory. There was a correlation between human-dependent transportation routes and the distribution of virus.

**Conclusion:**

Inter-regional migration paths of the viruses might be correlated with translocation of dogs associated with humans. Interconnecting factors between human socioeconomic and population density might determine the transmission dynamics of virus in a rural-to-urban polarity. The presence of 2 or more rabies virus groups in a location might be indicative of a gene flow, reflecting a translocation of dogs within such region and adjacent areas. Different approaches may be required for rabies control based on the homo- or heterogeneity of the virus. Areas containing homogeneous virus populations should be targeted first. Control of dog movement associated with humans is essential.

## Background

Rabies is not high on the list of the World Heath Organization's list of important infectious diseases, and is also often overlooked by regional, national, and local public-health professionals. The dog is the primary reservoir and vector of rabies transmission in Thailand and developing countries [1].

To date, the evaluation of the importance of rabies has been determined solely by estimating the number of human deaths and statistics on dog rabies infectivity, which may not be a reliable indicator in developing countries [2]. For example, an accurate assessment of the burden of rabies will never be complete without including the financial burden incurred due to human rabies post-exposure prophylaxis (PEP) and animal control.

In Thailand, the substantial decline in human rabies deaths from almost 200 a decade ago to less than 20 in 2003, has occurred due to the huge and continuously escalating financial obligation in the annual budget required to supply rabies biologicals for human PEP. More than 400,000 patients received PEP in 2003, as compared to approximately 90,000 in 1991 [Ministry of Public Health (MOPH) annual report]. Moreover, annual human rabies deaths in Bangkok, where diagnostic facilities and neurologists are readily available, rose from less than 5 in 1990–1994 to 5–10 in 1995–2001 (MOPH annual report).

There are no reliable statistical analyses of dog populations that could be evaluated to determine the effectiveness of the current human rabies prevention methodologies used in Thailand. One quoted figure of 6 million dogs in Thailand is undoubtedly an underestimate of the actual population present within the country. The Division of Disease Control and Ministry of Agriculture reported that between 60 to 78% of the dog population was vaccinated (based on estimated total population) in Thailand between 1995 and 2000. Experience in Latin America has shown that vaccination of a critical percentage of dogs, on the order of 40–70%, at least in major urban areas, was sufficient to interrupt canine rabies transmission and resulted in diminished human rabies deaths [3]. However, this has not been the case in Thailand. The percentage of rabies infectivity of samples sent to diagnostic laboratories all over the country remains high, within the range of 30–40% (MOPH annual report).

A survey in 1999 by the Department of Livestock and the Bangkok Metropolitan Administration revealed that stray dog populations in Bangkok (an area of 1,565 sq km) have tripled in size, (from 40,756 in 1992 to 110,584 in 1999). Additionally, a 2002 survey suggested that dog populations were increasing, both in Bangkok and the countrywide, implying that the specific carrying capacity of canine habitats has not yet been saturated. Moreover, a substantial number of dogs, especially stray and community dogs, are not vaccinated.

Due to budget limitation, an intensive dog vaccination and sterilization program has been in place only in Bangkok City since June 2002. Seventy-two million baht (approximately US$ 1,800,000) were spent during the first 2 phases of the program (June 2002-September 2003), with the third phase (October 2003-September 2004) costing an additional 31 million baht (approximately US$ 775,000). Although there were no human rabies deaths in Bangkok in 2002, 3 deaths were reported in 2003. Preliminary assessment revealed that less than 20 percent of the estimated dog population was sterilized and vaccinated.

Without reliable data on dog ecology and surveillance of rabies infection in dogs and humans, it is not possible to develop a strategic plan for rabies prevention and control and to assess program success. Therefore, our objective was to use molecular biological techniques to characterize the presence and movement of rabies virus according to geographical locations in Thailand and use this information as baseline data to design and implement rabies prevention programs in the country. Areas with evidence of continuous gene flow, and presence of viruses of more than one genetic group or subclade, were characterized. The potential translocation of rabies virus from one area to another was evaluated in relation to natural barriers, transportation routes, human activity and socioeconomic factors.

## Methods

### Samples

Two hundred and thirty nine brain samples (7 humans, 7 cats, 216 dogs, 6 cattle, 1 water buffalo, 2 squirrels) from 56 provinces were obtained from 25 diagnostic laboratories all over Thailand between 1998 and 2002. Samples selected for analysis were chosen to be representative of the geographical location in each province down to the scale of small districts (subdivisions of a province). Samples were not available from 20 provinces. All samples were prescreened for evidence of rabies virus using the direct fluorescence antibody test and kept frozen at -80 degree C until genetic analysis was conducted.

### Genetic analysis

Genetic typing was based on nucleotide sequence differences in cDNA obtained by direct one step RT-PCR amplification of the nucleocapsid (N) gene fragment from the samples. The amplified products of 414 bp (nt 1101 – 1506) were characterized by sequencing. RT-PCR and sequencing procedures were conducted as previously described [4]. One set of primers was used for RT-PCR sequencing reaction. GenBank accession numbers of the N sequences in this study were AY849022-AY849260 (see Additional file 1).

Twelve additional N sequences were retrieved from Genbank database to be outgroups for this study: a non-rabies lyssavirus Mokola Virus (S59448), 3 Australian Bat Lyssavirus (ABLV) isolates (NC003243, AF081020, AF418014), a rabies strain Pasteur Virus (PV) (M13215), 6 rabies viruses from other Asian countries (AY138550 from Sri Lanka, AY138551 Sri Lanka, AY138549 Sri Lanka, AF155039 China, AF374721 India, U22482 Iran) and 1 rabies isolate from Thailand (U22653). The sequences of all isolates were aligned together using program ClustalX [5]. Genetic relationships between these N gene sequences were calculated and a tree diagram was drawn using neighbor-joining (NJ) method, which was suitable to illustrate below species-level genetic relationships. These phylogenetic analyses were performed with program PAUP* version 4.0b10 [6]. Robustness of the tree was accessed with branch supporting-values from bootstrap (BS) statistic analyses (1,000 replicates). The collecting provinces and districts of all virus samples were mapped on the trees. Geographical locations of samples were mapped (Arcview 3.2, ESRI) and compared among the subgroups.

## Results

**Figure 1:**
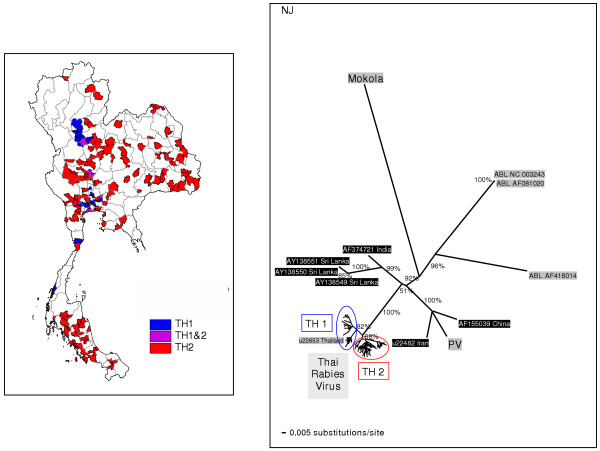
**Comparison between NJ tree of Thai rabies N genes and geographical distribution map**. Neighbor-joining (NJ) tree based on 414 bp nucleotide sequences of the N genes of all 239 Thai rabies virus isolates compared with other 11 lyssavirus outgroups. Numbers along tree branches are >50% bootstrap supporting-value (1,000 replicates). The map of Thailand indicates geographical distributions of the 2 major phylogroups, TH1 and TH2, in a district-level (a subdivision of a province).

Phylogenetic analyses of the 239 N rabies sequences collected from all over Thailand clearly demonstrated that all of the isolates formed a monophyletic group with 100% boostrap supporting values, separate from Mokola Virus, Australian Bat Lyssavirus (ABLV), Pasteur Virus (PV) and other Asian rabies viruses (Fig. 1). The N sequences from India and Sri Lanka were weakly grouped with those of Thai rabies virus with 51% bootstrap support. Neither the sequences of infected human nor non-canine animals (cats and other wildlife) were specifically clustered as a unique group, but rather paired with the dog rabies viruses analyzed in the study. Two major viral groups were clearly recognized from the tree and designated as TH1 and TH2 clusters, with 82% and 68% bootstrap supporting values, respectively.

**Figure 2:**
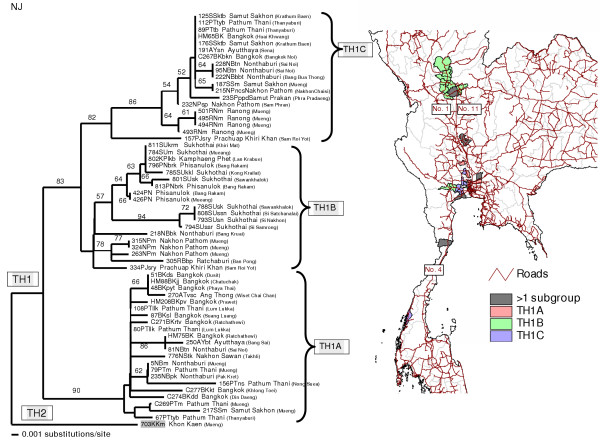
**Comparison between NJ tree of TH1 rabies sequences and the distribution map. **A comparison between the NJ tree of 60 N gene sequences of TH1 rabies viruses (with the TH2 isolate 703KKm added as an outgroup) and the Thailand map indicates geographical distributions of the subgroups TH1A, TH1B, and TH1C.

Considering sampling locations of viral isolates, both Thai rabies phylogroups were confined to certain geographical areas, though overlapping did occur in some areas (see the map of Thailand in Fig. 1). TH1 viruses were found mainly in the middle part of the country, from the lower northern region to the central region, Bangkok and surrounding provinces, and the upper southern region of Thailand. Based on BS supporting values on each branch, branch lengths (equally to numbers of substitutions/site), and the tree topology, the TH1 phylogroup was divided into 3 minor subgroups (Fig. 2): TH1A isolates were identified in Bangkok and outskirts as well as some other provinces in the central region; TH1B isolates were identified in the northern and central regions, and TH1C isolates were identified in Bangkok, the central, and the upper southern regions.

**Figure 3:**
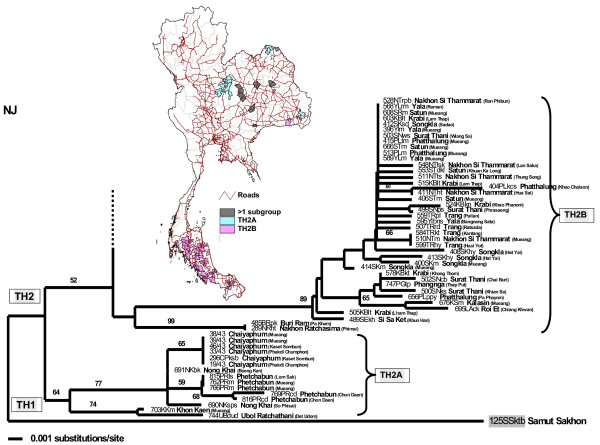
**Comparison between NJ tree of TH2A and TH2B rabies sequences and the distribution map. **A comparison between the bottom part of the NJ tree of TH2 rabies viruses (with the TH1 isolate 125SSktb added as an outgroup) and the Thailand map indicates geographical distributions of the subgroups TH2A and TH2B.

**Figure 4:**
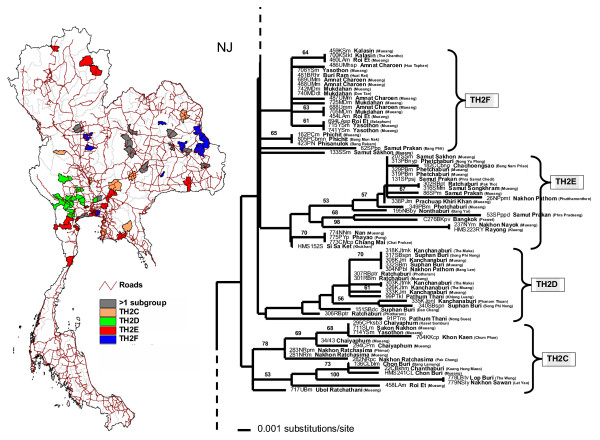
**Comparison between NJ tree of TH2C, TH2D, TH2E, and TH2F rabies sequences and the distribution map. **A comparison between the middle part of the NJ tree of TH2 rabies viruses and the Thailand map indicates geographical distributions of the subgroups TH2C, TH2D, TH2E, and TH2F.

**Figure 5:**
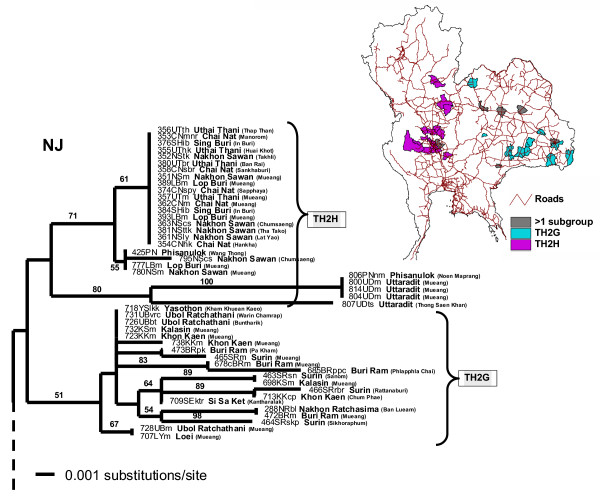
**Comparison between NJ tree of TH2G and TH2H rabies sequences and the distribution map. **A comparison between the top part of the NJ tree of TH2 rabies viruses and the Thailand map indicates geographical distributions of the subgroups TH2G and TH2H.

The TH2 rabies phylogroup was distributed in much wider areas than the TH1 phylogroup (Fig. 1), from the northern to the southern-most regions of the country. The distribution areas of TH2 group covered almost every province in the northeastern region, all main provinces in the upper central and the central regions, Bangkok and 5 surrounding provinces, the eastern and western regions, and nearly the entire area of southern Thailand. Using similar criteria as in the TH1 group, TH2 was divided into 8 subgroups: TH2A (Fig. 3) with samples from the northeastern region, TH2B (Fig. 3) with samples from the south and some northeastern provinces, TH2C (Fig. 4) from a few provinces scattered in the east, upper central, and northeast, TH2D (Fig. 4) from western provinces, TH2E (Fig. 4) had a much wider distribution-range including the far north, northeast, central including provinces around Bangkok, to the upper south, TH2F (Fig. 4) and TH2G (Fig. 5) were mainly located in the northeastern regions, and TH2H (Fig. 5) was found in the lower north to the upper central.

## Discussion

The use of molecular biological techniques to evaluate the epidemiology of viral diseases is being increasingly employed to complement conventional methods [[7-11], for examples of rabies epidemiology]. These techniques can give a clearer understanding of the origination and transmission patterns of viral epidemics. Eventually, data produced from molecular epidemiological studies could lead to a better understanding of and a more effective strategy to control the spread of infectious diseases.

Our study revealed that all of the currently identified Thai rabies viruses share a common origin that is genetically distant from the PV, ABLV, and Mokola outgroups. Additionally, the monophyletic tree of the Thai rabies viruses analyzed in this study was clearly distinguishable from other rabies N sequences from India, Sri Lanka, China, and Iran (Fig. 1). Thus, rabies viruses circulating in Thailand (or in Southeast Asia) could possibly have an exclusive evolutionary background that might be recognized as being unique, an hypothesis previously suggested by Susetya et al. [12]. It will be necessary to analyze additional sequences of rabies viruses circulating in neighboring countries adjacent to Thailand to confirm this hypothesis.

The NJ genetic distance tree also confirmed that the sequences obtained from non-canine sources (human, cats and other mammals) were very similar to those obtained from rabid dogs. No specific grouping of sequences from rabies virus isolated from non-canine species was identified. Instead, these rabies virus sequences were scattered across the tree. This finding was in accord to our expectation that the dog is a prime reservoir and transmitting vector for rabies and causes spillover to human and domestic animals and wildlife. Nevertheless, we are also aware that there may be other vectors, such as bats, and other lyssaviruses, besides genotype 1, circulating in Thailand. In fact, our recent survey in Thai bats indicated that as many as 7.5% of the bat population had evidence of lyssavirus infection by an as yet unidentified genotype(s) [13].

The 2 major groups found in our Thai rabies phylogeny were judged to be significant with high bootstrap supporting values. Notably, these 2 major lineages resembled the putative groups A and B found in our previous study [14] in which fewer numbers of samples from Bangkok and its surrounding provinces were analyzed. The 2 phylogroups we identified had certain trends in their geographical distributions. The distribution areas of TH1 group were only found in the central part of the country – from Nakhon Sawan province, down along Choa Praya river to the capital city of Bangkok, ending at Ranong province in the upper southern region (Fig. 2). On the other hand, those of TH2 group were spread across more than three-quarters of the entire country – from Phayao province in the north (Fig. 4), to Ubol Ratchathani in the northeast corner (Fig. 5), and to the southern Yala province along the Thailand-Malaysia border (Fig. 3).

Although there are some overlapping areas shared between the TH1 and TH2 phylogroups, the viral transmission dynamics and evolutionary background the sub-lineages may not be similar which could explain why both have different success levels in disease dispersals. It has been proposed that the degree of differences in compartmentalization mechanisms may influence the duration that each individual canine-associated rabies variant resides in certain geographical regions [15]. Relationships between dogs and humans within a community, dog population density, and relative dog-human population ratio are common explanations for such compartmentalization phenomenon [16,17]. Local geographical barriers such as rivers and mountains are other important factors considered to have strong influences on the inhibition of the spread of vector-borne diseases [18]. This inhibition effect caused by natural barriers could, however, be compromised by human transportation routes, for instance bridges, or roads and railways through mountains.

**Figure 6:**
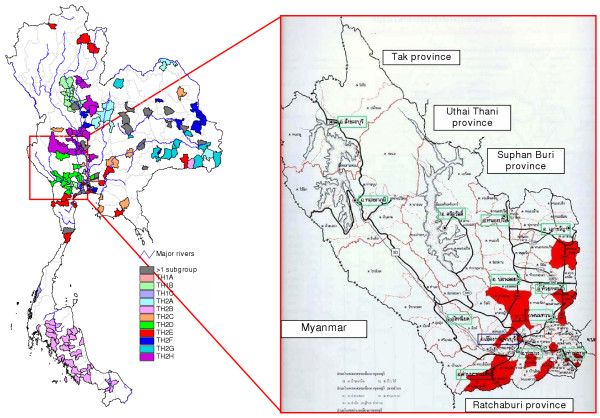
**Comparison between geographical distribution of rabies viruses in Thailand and in Kanchanaburi province. **Geographical distribution of all Thai rabies virus subgroups. Kanchanaburi province was magnified to show province geography and roadmap. Red areas in the Kanchanaburi map indicate the collecting localities of rabies hosts in a tambon (a subdivision of a district)-level. The map was retrieved from

To estimate the epidemiological characteristics of the TH1 and TH2 Thai rabies groups, a phylogeographical concept was introduced to infer their transmission dynamics [19,20]. Comparison between the sampling localities and molecular phylogenetic-tree topology could determine how viruses in each and different group are genetically related. First, in Bangkok and the surrounding provinces both TH1 and TH2 were identified as occurring together (Fig. 1). This area is industrialized and highly populated. Hundreds of mainroads, highways, and railways have been built in the country heartland. Networks of transportation routes including bridges across waterways are an effective means for vector borne viral transmission. This is one explanation as to why some viral subgroups of both TH1 and TH2 were discovered along both sides of Choa Phraya and many other rivers (Fig. 6), as has been previously reported [14].

Secondly, we suggest that transmission of rabies virus may be related to human activity, particularly human migration. Considering the phylogeographic areas of the 3 genetic subgroups of TH1 rabies virus (Fig. 2), genetic exchanges within the TH1B subgroup between Sukhothai in the north and Nonthaburi province near Bangkok, almost 500-kilometres apart, could not have been accomplished by migrations of animal virus-vectors alone. It is more likely that canine vectors of the TH1B genotype were translocated from areas around Bangkok to the north, and vice versa, simply by following movements of pet-owners via the national mainroads number 1 and 11 (Fig. 2). Similarly, the same translocation factors can be applied to a long-distance dispersal of the TH1C subgroup from central to southern Thailand, probably via the national mainroad number 4 (Fig. 2). Transmission dynamics of the TH1 subgroup might also have been influenced by a combination of factors including social and socioeconomic status, human and animal population density in addition to the availability of transportation routes.

The theory that the spread of canine rabies virus was instigated by pet-owner translocation via transportation routes was supported in this study by the results of the distribution pattern of each subgroup of TH2 (Figs. 3, 4, 5). Members of the TH2 group appeared to be scattered across the regions at a very distant range, and are unlikely to have occurred due to animal self-translocation. From our analyses, we propose that genetically linked viruses of each subgroup were localized in specific areas by utilizing transportation routes throughout Thailand (as shown in Fig. 3, 4, and 5), and areas that have more than one viral group present are apparently local transportation, for instance, Mueng district (Khon Kaen province), Pa Kham district (Buri Ram province) and Phimi district (Nakhon Ratchasima province) (shown as black areas in Fig. 3).

The most convincing support for the human-facilitated rabies distribution hypothesis we propose herein is the geographical distribution of the TH2B subgroup in which all, except a few samples from the northeast, were from the southern region of the country. This phylogeographic subgroup with a 1300–1600 km spreading range, had a very strong bootstrap supporting-value (89%) on the genetic tree. We propose that this inter-regional migration path of the TH2 subgroup is explained by a rural-to-urban viral transmission polarity. [12,16] The majority of people in the northeast have a relatively lower socioeconomic status than people living in other regions. Most of them are conventional crop farmers with low annual income [9,279 Baht (approximately US$ 230) average monthly household income versus national average of 13,736 Baht (approximately US$ 340), reported by National Statistical Office on 2002] and during the off-growing season they usually migrate to other regions to seek employment as common laborers. The strong economics in southern Thailand has been mainly supported by marine fishery as well as the rubber plant and oil palm agricultural industry, of which most workers originate from northeastern Thailand. Rabies virus infected canine pets accompanying the migratory workers from the northeastern therefore could be spread along their owners' travel routes. This would not only explain the northeast-to-south migration path of the TH2B viruses, but also could elucidate why most of the TH2 subgroups examined were closely linked with viral isolates from the northeastern region.

Selection of suitable areas using molecular epidemiological techniques should be considered as a powerful tool when planning disease control strategies. For decades, Thailand has invested vast sums of money and manpower on the effort to control and vaccinate the dog population in randomly selected districts and, recently, Bangkok capital city without success. Results of this research demonstrated that Bangkok and other metropolitan cities (such as Prathum Thani, Samut Sakhon, Nakhon Sawan, Khon Kaen, Ubol Ratchathani) contain various groups and subgroups of viruses, actively circulating to and from other surrounding provinces (Fig. 6). Therefore, developing a campaign for disease control in such city alone, without considering neighboring areas, is highly unlikely to be successful. We propose that the most appropriate place to initiate a rabies control campaign should be in a genetically isolated area, where there are either natural or artificial barriers to prevent further viral influxes.

On a national scale, we propose that rabies control can be successful if it is initiated in southern Thailand. This region contains only the TH2B rabies subgroup. Furthermore, it is an "island-like" area surrounding by Andaman Sea, Gulf of Thailand, and the Malaysian border. Influx from the TH1C subgroup has been restricted to an area around Ranong province, plausibly from high mountain ridges. Moreover, the majority of the population in southern Thailand are Muslims who do not keep pet dogs or feed stray dogs. Implementation of a rabies control in this region should therefore be effective in terms of a dog population reduction and vaccination campaign, and the enforcement of strict regulations regarding dog transfer.

In order to test this concept of "targeting a genetically defined area", a mass rabies control compaign should be conducted in a suitable-size province with a homogeneous virus population. The province of Kanchanaburi, 19,483 km sq and about 130 km westwards from Bangkok, appears to be a good choice since, according to our study, it contains only the TH2D rabies subgroups (Fig. 6) which are clustered mostly on the southernmost tambons (subdistricts; subdivisions of an district). The province is also island-like in that it is surrounded by the mountainous Thailand-Myanmar border and also has mountain ridges along the eastern boundary to other provinces. Any strategic plan in this region should also include recommendations to control pet-dog movements via the national mainroad number 323, the primary transportation route into the province. Moreover, in order to control rabies situation, at least 50 – 70% of dogs must be vaccinated. Currently available vaccine used in Thailand is injectable type, thus, requiring a capturing or restraining process which is extremely difficult especially in the case of community or stray dogs. Oral type vaccine such as that used in wildlife once proven of its safety and efficacy in dogs may be an alternative. Public participation in dog population control and vaccination needs to be created. Intensive and extensive educational activities should be carried out to increase understanding of the necessity to have rabies and dog population control program implemented [21]. Assessment of the success of such a program can be measured by a strict surveillance of rabies incidence in humans and animals and by analyzing genetic sequences of rabies virus as compared to others in adjacent provinces. This should also be correlated with transportation tracks on a local scale.

## Conclusion

In conclusion, we have presented a novel approach to the development of a rabies control and prevention program through the utilization of genetic epidemiology. We believe that the implementation of such a disease control program utilizing existing information on the genetics of circulating rabies viruses in a country like Thailand could be successful if the campaign target areas have been carefully selected and limited to one circulating phylogroup of virus and the movement of dogs along human transportation routes into the area is strictly enforced.

## Competing interests

The authors declare that they have no financial or personal relationships with other people or organizations that could inappropriately influence this research. All authors have access to all data in the study and held final responsibility for the decision to submit for publication.

## Authors' contributions

JD participated in data analysis and interpretation, phylogenetic tree construction, and writing the paper. SW participated in data collection and analysis, PCR primer design, sequencing rabies genes, and writing the paper. BL participated in specimen collection, data analysis, and writing the paper. NR participated in data analysis, figure preparation, and writing the paper. WH participated in coordinating the study with the veterinarians throughout the country, data analysis, and writing the paper. AP participated in coordinating the study with the physicians throughout the country, data analysis, and writing the paper. TH participated in study design, data analysis and interpretation, and writing the paper. The first 3 authors (JD, SW, BL) contributed equally to this work. All authors read and approved the final manuscript.

## Pre-publication history

The pre-publication history for this paper can be accessed here:



## Supplementary Material

Additional File 1 Table 1 Taxon list of N-gene sequences of Thai rabies virus used in this study Click here for file
