# Supplementary material for: Transmission dynamics of rabies virus in Thailand: Implications for disease control
Source: BMC Infect Dis. 2005 Jun 29;5:52. doi: 10.1186/1471-2334-5-52 (PMC1184074; doi:10.1186/1471-2334-5-52)
Supplement: Additional File 1 — Table 1 Taxon list of N-gene sequences of Thai rabies virus used in this study [file 1471-2334-5-52-S1.doc]

**Table 1** Taxon list of N-gene sequences of Thai rabies virus used in this study with GenBank accession numbers, viral isolation number, host species, collecting locality, and collecting year of isolation,

| **GenBank Accession no.** | **Isolation number** | **Species** | **Locality (district/province)** | **Year** |
| --- | --- | --- | --- | --- |
| AY849022 | 5NBm | Dog | Mueang/Nonthaburi | 2001 |
| AY849023 | 26NPpmt | Dog | Phutthamonthon/Nakhon Pathom | 2001 |
| AY849024 | 53SPppd | Dog | Phra Pradaeng/Samut Prakan | 1999 |
| AY849025 | 67PTtyb | Dog | Thanyaburi/Pathum Thani | 1999 |
| AY849026 | 80PTllk | Dog | Lum Lukka/Pathum Thani | 1999 |
| AY849027 | 81NBtn | Dog | Sai Noi/Nonthaburi | 2001 |
| AY849028 | 99PTkl | Dog | Khlong Luang/Pathum Thani | 1999 |
| AY849029 | 79PTm | Dog | Mueang/Pathum Thani | 1999 |
| AY849030 | 125SSktb | Dog | Krathum Baen/Samut Sakhon | 1999 |
| AY849031 | 133SSm | Dog | Mueang/Samut Sakhon | 1999 |
| AY849032 | 176SSktb | Dog | Krathum Baen/Samut Sakhon | 1999 |
| AY849033 | 187SSm | Dog | Mueang/Samut Sakhon | 1999 |
| AY849034 | 207SSm | Dog | Mueang/Samut Sakhon | 1999 |
| AY849035 | 217SSm | Dog | Mueang/Samut Sakhon | 1999 |
| AY849036 | 218NBbk | Dog | Bang Kruai/Nonthaburi | 2001 |
| AY849037 | 222NBbbt | Dog | Bang Bua Thong/Nonthaburi | 1999 |
| AY849038 | 228NBtn | Dog | Sai Noi/Nonthaburi | 1999 |
| AY849039 | 304NPbl | Dog | Bang Len/Nakhon Pathom | 2001 |
| AY849040 | 315NPm | Dog | Mueang/Nakhon Pathom | 2001 |
| AY849041 | 324NPm | Dog | Mueang/Nakhon Pathom | 2001 |
| AY849042 | 91PTns | Dog | Nong Suea/Pathum Thani | 1999 |
| AY849043 | 95NBtn | Dog | Sai Noi/Nonthaburi | 1999 |
| AY849044 | 108PTllk | Dog | Lum Lukka/Pathum Thani | 1999 |
| AY849045 | 112PTtyb | Dog | Thanyaburi/Pathum Thani | 1999 |
| AY849046 | 195NBby | Dog | Bang Yai/Nonthaburi | 1999 |
| AY849047 | 215NPncs | Dog | Nakhon Chaisi/Nakhon Pathom | 1999 |
| AY849048 | 232NPsp | Dog | Sam Phran/Nakhon Pathom | 1999 |
| AY849049 | 235NBpk | Dog | Pak Kret/Nonthaburi | 1999 |
| AY849050 | 263NPm | Dog | Mueang/Nakhon Pathom | 1999 |
| AY849051 | C267BKbkn | Cat | Bangkok Noi/Bangkok | 2000 |
| AY849052 | C269PTm | Cat | Mueang/Pathum Thani | 2000 |
| AY849053 | C271BKrtv | Cat | Ratchathewi/Bangkok | 2001 |
| AY849054 | C274BKdd | Cat | Din Daeng/Bangkok | 1999 |
| AY849055 | C276BKpv | Cat | Prawet/Bangkok | 1999 |
| AY849056 | C277BKkt | Cat | Khlong Toei/Bangkok | 1999 |
| AY849057 | 303KJtmk | Dog | Tha Maka/Kanchanaburi | 2001 |
| AY849058 | 308KJm | Dog | Mueang/Kanchanaburi | 2001 |
| AY849059 | 318KJtmk | Dog | Tha Maka/Kanchanaburi | 2001 |
| AY849060 | 326KJtm | Dog | ThaMuang/Kanchanaburi | 2001 |
| AY849061 | 333KJm | Dog | Mueang/Kanchanaburi | 2001 |
| AY849062 | 335KJpnt | Dog | Phanom Thuan/Kanchanaburi | 2001 |
| AY849063 | 89PTtb | Dog | Thanyaburi/Pathum Thani | 1999 |
| AY849064 | 38/43 | Dog | Mueng/Chaiyaphum | 2000 |
| AY849065 | 39/43 | Dog | Mueng/Chaiyaphum | 2000 |
| AY849066 | 46/43 | Dog | Kaset Sombun/Chaiyaphum | 2000 |
| AY849067 | 23SPppd | Dog | Phra Pradaeng/Samut Prakan | 2001 |
| AY849068 | 33/43 | Dog | Phakdi Chumphon/Chaiyaphum | 2000 |
| AY849069 | 34/43 | Dog | Mueang/Chaiyaphum | 2000 |
| AY849070 | 19/43 | Dog | Phakdi Chumphon/Chaiyaphum | 2000 |
| AY849071 | 51BKds | Dog | Dusit/Bangkok | 1999 |

Table 1. (continue)

| **GenBank Accession no.** | **Isolation number** | **Species** | **Locality (district/province)** | **Year** |
| --- | --- | --- | --- | --- |
| AY849072 | 62SPbp | Dog | Bang Phli/Samut Prakan | 1999 |
| AY849073 | 48BKpyt | Dog | Phaya Thai/Bangkok | 2001 |
| AY849074 | HM65BK | Human | Huai Khwang/Bangkok | 1998 |
| AY849075 | HM75BK | Human | Ratchathewi/Bangkok | 1998 |
| AY849076 | HM88BKjj | Human | Chatuchak/Bangkok | 1999 |
| AY849077 | HM208BKpv | Human | Prawet/Bangkok | 2001 |
| AY849078 | 404PLkcs | Cattle | Khao Chaison/Phatthalung | 2002 |
| AY849079 | 355UThk | Dog | Huai Khot/Uthai Thani | 2001 |
| AY849080 | 415PLtm | Dog | Mueang/Phatthalung | 2002 |
| AY849081 | 294CPm | Dog | Mueang/Chaiyaphum | 2002 |
| AY849082 | 295CPksb3 | Dog | Kaset Sombun/Chaiyaphum | 2002 |
| AY849083 | 351NSm | Dog | Mueang/Nakhon Sawan | 2001 |
| AY849084 | 353CNmnr | Dog | Manorom/Chai Nat | 2001 |
| AY849085 | 363NScs | Dog | Chumsaeng/Nakhon Sawan | 2001 |
| AY849086 | 412SKsd | Dog | Sadao/Songkla | 2002 |
| AY849087 | 414SKm | Dog | Mueng/Songkla | 2002 |
| AY849088 | 361NSly | Dog | Lat Yao/Nakhon Sawan | 2001 |
| AY849089 | 376SHib | Dog | In Buri/Sing Buri | 2001 |
| AY849090 | 380UTbr | Dog | Ban Rai/Uthai Thani | 2001 |
| AY849091 | 393LBm | Dog | Mueang/Lop Buri | 2001 |
| AY849092 | 384SHib | Dog | In Buri/Sing Buri | 2001 |
| AY849093 | 411NTht | Dog | Hua Sai/Nakhon Si Thammarat | 2002 |
| AY849094 | 374CNspy | Dog | Sapphaya/Chai Nat | 2001 |
| AY849095 | 362CNm | Dog | Mueang/Chai Nat | 2001 |
| AY849096 | 357UTm | Dog | Mueang/Uthai Thani | 2001 |
| AY849097 | 356UTth | Dog | Thap Than/Uthai Thani | 2001 |
| AY849098 | 381NSttk | Dog | Tha Tako/Nakhon Sawan | 2001 |
| AY849099 | 408SKhy | Dog | Yai/Songkla | 2002 |
| AY849100 | 332SBm | Dog | Mueang/Suphan Buri | 2001 |
| AY849101 | 354CNhk | Dog | Hankha/Chai Nat | 2001 |
| AY849102 | 425PN | Dog | Wang Thong/Phisanulok | 2002 |
| AY849103 | 389LBm | Dog | Mueang/Lop Buri | 2001 |
| AY849104 | 423PN | Dog | Bang Rakam/Phisanulok | 2002 |
| AY849105 | 424PN | Dog | Bang Rakam/Phisanulok | 2002 |
| AY849106 | 413SKhy | Cattle | Yai/Songkla | 2001 |
| AY849107 | 358CNsbr | Dog | Sankhaburi/Chai Nat | 2001 |
| AY849108 | 317SBspn | Dog | Song Phi Nong/Suphan Buri | 2001 |
| AY849109 | 334PJsry | Dog | Sam Roi Yot/Prachuap Khiri Khan | 2001 |
| AY849110 | 340SBspn | Dog | Song Phi Nong/Suphan Buri | 2001 |
| AY849111 | 400SKm | Dog | Mueng/Songkla | 2001 |
| AY849112 | 87BKsl | Dog | Suang Luang/Bangkok | 2001 |
| AY849113 | 156PTns | Dog | Nong Suea/Pathum Thani | 1999 |
| AY849114 | 182CCbnp | Dog | Bang Nam Priao/Chachoengsao | 1999 |
| AY849115 | 349PBm | Dog | Mueang/Phetchaburi | 2001 |
| AY849116 | 307RBptr | Dog | Photharam/Ratchaburi | 2001 |
| AY849117 | 329PBm | Dog | Mueang/Phetchaburi | 2001 |
| AY849118 | 406STm | Dog | Mueang/Satun | 2002 |
| AY849119 | 352NStk | Dog | Takhli/Nakhon Sawan | 2001 |
| AY849120 | 301RBm | Dog | Mueang/Ratchaburi | 2001 |
| AY849121 | 157PJsry | Dog | Sam Roi Yot/Prachuap Khiri Khan | 1999 |
| AY849122 | 237NYm | Dog | Mueang/Nakhon Nayok | 1999 |
| AY849123 | 270ATvsc | Cat | Wiset Chai Chan/Ang Thong | 2001 |
| AY849124 | 281NRm | Dog | Mueang/Nakhon Ratchasima | 2001 |
| AY849125 | 282NRpc | Dog | Pak Chong/Nakhon Ratchasima | 2001 |
| AY849126 | 305RBbp | Dog | Ban Pong/Ratchaburi | 2001 |

Table 1. (continue)

| **GenBank Accession no.** | **Isolation number** | **Species** | **Locality (district/province)** | **Year** |
| --- | --- | --- | --- | --- |
| AY849127 | 306RBptr | Dog | Photharam/Ratchaburi | 2001 |
| AY849128 | 191AYsn | Dog | Sena/Ayutthaya | 1999 |
| AY849129 | 151SBdc | Dog | Dan Chang/Suphan Buri | 1999 |
| AY849130 | 22CBkhm | Dog | Kaeng Hang Maeo/Chanthaburi | 2001 |
| AY849131 | 162PCm | Dog | Mueang/Phichit | 1999 |
| AY849132 | 319PBm | Dog | Mueang/Phetchaburi | 2001 |
| AY849133 | 86SPm | Dog | Mueang/Samut Prakan | 2001 |
| AY849134 | 136CLblm | Dog | Bang Lamung/Chon Buri | 1999 |
| AY849135 | 396YLm | Dog | Mueang/Yala | 2001 |
| AY849136 | 250AYbt | Dog | Bang Sai/Ayutthaya | 1999 |
| AY849137 | 302RBpt | Dog | Pak Tho/Ratchaburi | 2001 |
| AY849138 | 316SMm | Dog | Mueang/Samut Songkhram | 2001 |
| AY849139 | 426PN | Dog | Mueang/Phisanulok | 2002 |
| AY849140 | 296CPksb | Dog | Kaset Sombun/Chaiyaphum | 2002 |
| AY849141 | 313PBnyp | Dog | Nong Ya Plong/Phetchaburi | 2001 |
| AY849142 | 288NRbl | Dog | Ban Lueam/Nakhon Ratchasima | 2002 |
| AY849143 | 505KBlt | Dog | Lham Thap/Krabi | 2000 |
| AY849144 | 507TRrd | Dog | Ratsada/Trang | 2000 |
| AY849145 | 511NTts | Dog | Thung Song/Nakhon Si Thammarat | 2000 |
| AY849146 | 515KBlt | Dog | Lam Thap/Krabi | 2000 |
| AY849147 | 524KBkn | Dog | Khao Phanom/Krabi | 2000 |
| AY849148 | 559TRpl | Cattle | Palian/Trang | 2000 |
| AY849149 | 578KBkt | Dog | Khong Thom/Krabi | 2001 |
| AY849150 | 584TRkt | Dog | Kantang/Satun | 2001 |
| AY849151 | 603KBlt | Dog | Lam Thap/Krabi | 2001 |
| AY849152 | 595Ylbns | Dog | Bangnang Sata/Yala | 2001 |
| AY849153 | 493RNm | Dog | Mueang/Ranong | 2000 |
| AY849154 | 656PLppy | Dog | Pa Phayom/Phatthalung | 2002 |
| AY849155 | 676KSm | squirrel | Mueng/Kalasin | 2002 |
| AY849156 | 513PLm | Dog | Mueang/Phatthalung | 2000 |
| AY849157 | 589YLm | Dog | Mueang/Yala | 2001 |
| AY849158 | 608SRm | Dog | Mueang/Satun | 2001 |
| AY849159 | HMS152S | Human | Khunkhan/Si Sa Ket | 2001 |
| AY849160 | 599TRhy | Dog | Huai Yot/Trang | 2001 |
| AY849161 | 473BRpk | Dog | Pa Kham/Surin | 2001 |
| AY849162 | 485BRpk | Dog | Pa Kham/Buri Ram | 2002 |
| AY849163 | 510NTm | Dog | Mueang/Nakhon Si Thammarat | 2000 |
| AY849164 | 486UMhsp | Dog | Hua Taphan/Amnat Chareon | 2002 |
| AY849165 | 487UMm | Dog | Mueang/Amnat Charoen | 2002 |
| AY849166 | 501RNm | Dog | Mueang/Nakhon Si Thammarat | 2002 |
| AY849167 | 283NRpm | Dog | Phimai/Nakhon Ratchasima | 2001 |
| AY849168 | 459KSm | Dog | Mueang/Kalasin | 2002 |
| AY849169 | 463SRsn | Dog | Sanom/Surin | 2001 |
| AY849170 | 464SRskp | Dog | Sikhoraphum/Surin | 2001 |
| AY849171 | 465SRm | Dog | Mueang/Surin | 2001 |
| AY849172 | 466SRrbr | Dog | Rattanaburi/Surin | 2001 |
| AY849173 | 528NTrpb | Dog | Ron Phibun/Nakhon Si Thammarat | 2000 |
| AY849174 | 548NTlsk | Dog | Lan Saka/Nakhon Si Thammarat | 2000 |
| AY849175 | 289NRht | Dog | Phimai/Nakhon Ratchasima | 2001 |
| AY849176 | 723KKm | Dog | Mueang/Khon Kaen | 2001 |
| AY849177 | 481BRhr | Dog | Huai Rat/Buri Ram | 2002 |
| AY849178 | 494RNm | Dog | Mueang/Ranong | 2000 |
| AY849179 | 503SNws | Dog | Wiang Sa/Surat Thani | 2002 |
| AY849180 | 553STdkl | Dog | Khuan Ka Long/Satun | 2000 |

Table 1. (continue)

| **GenBank Accession no.** | **Isolation number** | **Species** | **Locality (district/province)** | **Year** |
| --- | --- | --- | --- | --- |
| AY849181 | 666STm | Cattle | Mueang/Satun | 2002 |
| AY849182 | 458LAm | Dog | Mueang/RoiEt | 2001 |
| AY849183 | 500SNks | Dog | Khian Sa/Surat Thani | 2002 |
| AY849184 | 454LAm | Dog | Mueang/Roi Et | 2001 |
| AY849185 | 495RNm | Dog | Mueang/Ranong | 2001 |
| AY849186 | 460LAm | Dog | Mueang/Roi Et | 2002 |
| AY849187 | 488UMm | Dog | Mueang/Amnat Charoen | 1998 |
| AY849188 | 489SEkh | Dog | Khun Han/Si Sa Ket | 2000 |
| AY849189 | 472BRm | Dog | Mueang/Buri Ram | 2001 |
| AY849190 | 502SNcb | Dog | Chai Buri/Surat Thani | 2002 |
| AY849191 | 499SNps | Dog | Phrasaeng/Surat Thani | 2002 |
| AY849192 | HMS241CL | Human | Mueang/Chon Buri | 2001 |
| AY849193 | 694LAsp | Dog | Selaphum/Roi Et | 2002 |
| AY849194 | 690NKsps | Dog | So Phaisai/Nong Khai | 2002 |
| AY849195 | 689Umm | Dog | Mueang/Amnat Charoen | 2001 |
| AY849196 | 688Umm | Dog | Mueang/Amnat Charoen | 2002 |
| AY849197 | 691NKbk | Dog | Bueng Kan/Nong Khai | 2002 |
| AY849198 | 695LAck | Dog | Chiang Khwan/Roi Et | 2002 |
| AY849199 | 698KSm | squirrel | Mueang/Kalasin | 2002 |
| AY849200 | 700KStkt | Dog | Tha Khantho/Kalasin | 2003 |
| AY849201 | 708YSm | Dog | Mueang/Yasothon | 2000 |
| AY849202 | 568YLrm | Dog | Raman/Yala | 2000 |
| AY849203 | 725MDm | Dog | Mueang/Mukdahan | 2001 |
| AY849204 | 726UBbt | Dog | Buntharik/Ubol Ratchathani | 2001 |
| AY849205 | 738KKm | Dog | Mueang/Khon Kaen | 2002 |
| AY849206 | 742MDm | Dog | Mueang/Mukdahan | 2002 |
| AY849207 | 740MDdt | Dog | Don Tan/Mukdahan | 2002 |
| AY849208 | 711SLm | Dog | Mueang/Sakon Nakhon | 2000 |
| AY849209 | 709SEktr | Dog | Kantharalak/Si Sa Ket | 2000 |
| AY849210 | 678cBRm | Cat | Mueang/Buri Ram | 2000 |
| AY849211 | 728UBm | Cattle | Mueang/Ubol Ratchathani | 2001 |
| AY849212 | 685BRppc | Dog | Phlapphla Chai/Buri Ram | 2002 |
| AY849213 | 714YSm | Dog | Mueang/Yasothon | 2001 |
| AY849214 | 715YSm | Water buffalo | Mueang/Yasothon | 2001 |
| AY849215 | 718YSlkk | Dog | Kham Khuean Kaeo/Yasothon | 2001 |
| AY849216 | 705MDm | Dog | Mueang/Mukdahan | 2000 |
| AY849217 | 731UBvrc | Dog | Warin Chamrap/Ubol Ratchathani | 2001 |
| AY849218 | 704KKcp | Dog | Chum Phae/Khon Kaen | 2000 |
| AY849219 | 703KKm | Dog | Mueang/Khon Kaen | 2000 |
| AY849220 | 732KSm | Cattle | Mueang/Kalasin | 2001 |
| AY849221 | 741YSm | Dog | Mueang/Yasothon | 2002 |
| AY849222 | 744UBdud | Dog | Det Udom/Ubol Ratchathani | 2002 |
| AY849223 | 713KKcp | Dog | Chum Phae/Khon Kaen | 2001 |
| AY849224 | 717UBm | Dog | Mueang/Ubol Ratchathani | 2001 |
| AY849225 | 707LYm | Dog | Mueang/Loei | 2000 |
| AY849226 | 131SPpsj | Dog | Phra Samut Chedi/Samut Prakan | 1999 |
| AY849227 | HMS223RY | Human | Klaeng/Rayong | 2002 |
| AY849228 | 747PGtp | Dog | Thap Put/Phangnga | 2003 |
| AY849229 | 766PRm | Dog | Mueang/Phetchabun | 2003 |
| AY849230 | 769PRcd | Dog | Chon Daen/Phetchabun | 2003 |
| AY849231 | 773CMcp | Dog | Chai Prakan/Chiang Mai | 2002 |

Table 1. (continue)

| **GenBank Accession no.** | **Isolation number** | **Species** | **Locality (district/province)** | **Year** |
| --- | --- | --- | --- | --- |
| AY849232 | 774NNm | Dog | Mueang/Nan | 2002 |
| AY849233 | 775PYp | Dog | Pong/Phayao | 2003 |
| AY849234 | 776NStk | Dog | Takhli/Nakhon Sawan | 2002 |
| AY849235 | 795NScs | Dog | Chumsaeng/Nakhon Sawan | 2003 |
| AY849236 | 801SUsk | Dog | Sawakhalok/Sukhothai | 2002 |
| AY849237 | 802KPlkb | Dog | Lan Krabue/Kamphaeng Phet | 2002 |
| AY849238 | 811SUkrm | Dog | Khiri Mat/Sukhothai | 2002 |
| AY849239 | 806PNnm | Dog | Noen Maprang/Phisanulok | 2002 |
| AY849240 | 814UDm | Dog | Mueang/Uttaradit | 2002 |
| AY849241 | 807UDts | Dog | Thong Saen Khan/Uttaradit | 2002 |
| AY849242 | 800Udm | Dog | Mueang/Uttaradit | 2002 |
| AY849243 | 815PRls | Dog | Lom Sak/Phetchabun | 2002 |
| AY849244 | 805PCbmn | Dog | Bang Mun Nak/Phichit | 2002 |
| AY849245 | 780NSm | Dog | Mueang/Nakhon Sawan | 2003 |
| AY849246 | 762PRm | Dog | Mueang/Phetchabun | 2003 |
| AY849247 | 813PNbrk | Dog | Bang Rakam/Phisanulok | 2002 |
| AY849248 | 796PNbrk | Dog | Bang Rakam/Phisanulok | 2003 |
| AY849249 | 816PRcd | Dog | Chon Daen/Phetchabun | 2002 |
| AY849250 | 778LBtv | Dog | Tha Wung/Lop Buri | 2003 |
| AY849251 | 794SUssr | Dog | Si Samrong/Sukhothai | 2003 |
| AY849252 | 784SUm | Dog | Mueang/Sukhothai | 2003 |
| AY849253 | 785SUkkl | Dog | Kong Krailat/Sukhothai | 2003 |
| AY849254 | 788SUsk | Dog | Sawankhalok/Sukhothai | 2003 |
| AY849255 | 808SUssn | Dog | Si Satchanalai/Sukhothai | 2002 |
| AY849256 | 793SUsn | Dog | Si Nakhon/Sukhothai | 2003 |
| AY849257 | 777LBm | Dog | Mueang/Lop Buri | 2003 |
| AY849258 | 779NSly | Dog | Lat Yao/Nakhon Sawan | 2003 |
| AY849259 | 804UDm | Dog | Mueang/Uttaradit | 2002 |
| AY849260 | 338PJm | Dog | Mueang/Prachuap Khiri Khan | 2001 |
